# The Molecular Basis for Oat Intolerance in Patients with Celiac Disease

**DOI:** 10.1371/journal.pmed.0010001

**Published:** 2004-10-19

**Authors:** Helene Arentz-Hansen, Burkhard Fleckenstein, Øyvind Molberg, Helge Scott, Frits Koning, Günther Jung, Peter Roepstorff, Knut E. A Lundin, Ludvig M Sollid

**Affiliations:** 1Institute of Immunology, Rikshospitalet University Hospital, University of OsloOsloNorway; **2**Department of Biochemistry and Molecular Biology, University of Southern DenmarkOdenseDenmark; **3**Institute of Pathology, Rikshospitalet University Hospital, University of OsloOsloNorway; **4**Department of Immunohematology and Blood Transfusion, Leiden University Medical CentreLeidenNetherlands; **5**Institute of Organic Chemistry, University of TübingenTübingenGermany; **6**Department of Medicine, Rikshospitalet University HospitalOsloNorway; University College LondonUnited Kingdom

## Abstract

**Background:**

Celiac disease is a small intestinal inflammatory disorder characterized by malabsorption, nutrient deficiency, and a range of clinical manifestations. It is caused by an inappropriate immune response to dietary gluten and is treated with a gluten-free diet. Recent feeding studies have indicated oats to be safe for celiac disease patients, and oats are now often included in the celiac disease diet. This study aimed to investigate whether oat intolerance exists in celiac disease and to characterize the cells and processes underlying this intolerance.

**Methods and Findings:**

We selected for study nine adults with celiac disease who had a history of oats exposure. Four of the patients had clinical symptoms on an oats-containing diet, and three of these four patients had intestinal inflammation typical of celiac disease at the time of oats exposure. We established oats-avenin-specific and -reactive intestinal T-cell lines from these three patients, as well as from two other patients who appeared to tolerate oats. The avenin-reactive T-cell lines recognized avenin peptides in the context of HLA-DQ2. These peptides have sequences rich in proline and glutamine residues closely resembling wheat gluten epitopes. Deamidation (glutamine→glutamic acid conversion) by tissue transglutaminase was involved in the avenin epitope formation.

**Conclusions:**

We conclude that some celiac disease patients have avenin-reactive mucosal T-cells that can cause mucosal inflammation. Oat intolerance may be a reason for villous atrophy and inflammation in patients with celiac disease who are eating oats but otherwise are adhering to a strict gluten-free diet. Clinical follow-up of celiac disease patients eating oats is advisable.

## Abstract 2

Are oats safe for patients with celiac disease? Some patients studied here have an immune response to the oat protein avenin and show disease symptoms

## Introduction

Celiac disease is a chronic inflammatory condition caused by an inappropriate immune response of intestinal T-cells reactive to gluten proteins of wheat and similar prolamin proteins of related cereals [1]. The majority of the peptides recognized by intestinal T-cells are more immunogenic following deamidation by tissue transglutaminase (TG2). These peptides are invariably presented by HLA-DQ2 or -DQ8, the same HLA molecules that confer genetic predisposition to celiac disease [1]. Gluten-reactive intestinal T-cells can be isolated from virtually all patients with celiac disease but not from normal individuals. The disease goes into remission when harmful cereals are avoided. A gluten-free diet is thus the standard treatment of this disorder.

Oats have traditionally been excluded from the gluten-free diet. Several feeding studies, however, have indicated that patients with celiac disease and dermatitis herpetiformis tolerate oats without signs of intestinal inflammation [2–9]. Of note, some of these studies have high patient-dropout rates that may have masked cases of oat intolerance. An in vitro study found no signs of T-cell activation in small intestinal biopsies of celiac disease patients challenged with avenin (the prolamin fraction of oats) [10], and avenins have been predicted to contain only a few glutamines that can be deamidated by TG2, presumably making avenins less immunogenic [11,12]. On this basis, oats have been allowed in the gluten-free diet in several countries [13].

It remains to be proven that all celiac disease patients tolerate oats following long-term exposure. A recent study of 39 Finnish patients randomized to eat a gluten-free diet with 50 g of oats daily or a standard gluten-free diet for 1 y reported more intestinal symptoms and more gut inflammation in the group of patients eating oats, although the mucosal integrity was not disturbed [14]. In an open challenge study of 19 adult celiac disease patients using pure oats, one patient developed villous atrophy [15]. This finding prompted us to investigate the phenomenon of oat intolerance further in a selected series of nine adult celiac disease patients, three of whom had clinical oat intolerance. The goal of the study was to characterize the intestinal T-cell response to oats avenin proteins in these patients in detail and to relate this to clinical symptoms and intestinal biopsy findings.

## Methods

### Participants

We studied nine adults with celiac disease who had a history of exposure to pure oats. The oats were derived from a quality-controlled production line and were shown to be free from contamination of other cereals as described elsewhere [15]. The selection of the study participants was not random. Five of the patients (CD359, CD377, CD422, CD431, and CD482) participated in a clinical challenge study consisting of 19 adults with celiac disease who ate 50 g oats daily for 12 wk [15]. One of these patients (CD422) has symptoms and mucosal inflammation on oats consumption as described [15]. Patient CD431 has slight mucosal inflammation when eating oats but is clinically well. The three remaining individuals eat and tolerate oats. All these five patients agreed to undergo gastroduodenoscopy for research purposes. In addition, two other adults with celiac disease (CD446 and CD504) were recruited from our ordinary outpatient clinic. Patient CD446 eats and tolerates oats, whereas patient CD504 has anaphylactoid symptoms after intake of oats but has no mucosal inflammation. Finally, two patients (CD496 and CD507) were referred by a general practitioner and a referring hospital for investigation of complications arising when eating a gluten-free diet, here termed complicated celiac disease. The latter four patients came for gastroduodenoscopy for clinical reasons, and agreed to have extra biopsies taken for research purposes. We were unable to measure serological parameters in these last four patients because no serum samples were taken from them during their clinical course. The study was approved by the regional ethical committee. The participants gave their informed consent.

### Histopathological Assessment

We took small intestinal biopsies from the horizontal part of the duodenum by gastroduodenoscopy using an Olympus (Tokyo, Japan) GIF-IT140 scope and scored them according to the modified Marsh criteria [16]. Intraepithelial lymphocytes were counted in hematoxilin-eosin-stained sections and enumerated per 100 enterocytes. Five areas per biopsy were counted, each encompassing 50–100 epithelial cells.

### Grain Antigens and Peptides

Oat grains (Regal, Oslo, Norway) were ground and the flour was washed twice with water-saturated 1-butanol. The pellet was dissolved in 45% ethanol overnight and centrifuged. The avenin fraction was precipitated from the supernatant by adding two volumes of 1.5 M NaCl. The precipitate was dissolved either in 0.01 M acetic acid (pH 1.8) and digested with pepsin and subsequently trypsin (pH 7.8) or in 2 M urea /0.01 M NH_4_HCO_3_ and digested with chymotrypsin. Gluten and gliadin (ethanol-soluble proteins of gluten) were isolated from household wheat flour and digested with chymotrypsin as described [17].

Avenin peptides were synthesized on a robotic system (Syro MultiSynTech, Bochum, Germany) using Fmoc/OtBu chemistry and 2-chlorotrityl resin (Senn Chemicals, Dielsdorf, Switzerland). The identity of the peptides was confirmed by electrospray mass spectrometry, and purity was analyzed by reverse-phase HPLC.

Treatment of the peptides with guinea pig (Sigma; St. Louis, Missouri, United States) and human recombinant TG2 was performed in the presence of 1 and 2 mM CaCl_2_, respectively, at 37 °C for 2 h.

### T-Cell Assays

The generation of T-cell lines, T-cell cloning, and T-cell proliferation assays were performed as described elsewhere [17]. Single biopsy specimens from the patients were challenged in vitro with either a pepsin-trypsin digest or a chymotrypsin digest of avenin. As control, single biopsy specimens were challenged with a chymotrypsin digest of gluten or gliadin. DR3+DQ2+ B lymphoblastoid cells (irradiated 80 Gy) were used as antigen-presenting cells. Positive T-cell responses were defined as a stimulatory index (SI) ([T + APC + antigen] divided by [T + APC]) above 3. Determination of HLA restriction of the T-cells was done by testing inhibition of T-cell proliferation by the monoclonal antibodies B8.11 (anti-DR), SPV-L3 (anti-DQ), 2.12.E11 (anti-DQ2), and W6/32 (anti-HLA class I).

### Purification of Avenin Fragments

A pepsin-trypsin digest of avenin was separated by gel filtration (FPLC, column Superdex Peptide HR 10/30; Pharmacia Biotech, Uppsala, Sweden), and a fraction containing T-cell stimulatory fragments was further separated by reverse-phase HPLC (Äkta, Pharmacia Biotech; column Jupiter 5μ C18, 250 × 4.6 mm, Phenomenex, Torrance, California, United States) using an acetonitrile gradient from 5% to 40% with 1%/min and from 40% to 64% with 3%/min (flow rate 0.9 ml/min, containing 0.05% trifluoroacetic acid).

### Mass Spectrometry and Database Searching

Electrospray ionization tandem mass spectrometry was performed on a quadrupole time-of-flight hybrid mass spectrometer (Micromass, Manchester, England). For spraying, needles were typically held at 900 V towards a skimmer cone (40 V). In collision-induced dissociation of selected peptide ions (collision gas argon; collision energy 25–35 eV), the generated characteristic b- and y-type fragment ions [18] were detected by the orthogonal TOF mass analyzer. All tandem mass spectrometry spectra were centroided and searched against in the NCBInr database via the Mascot Search Engine (http://www.matrixscience.com).

## Results

### Clinical and Histological Characteristics

**Figure 1:**
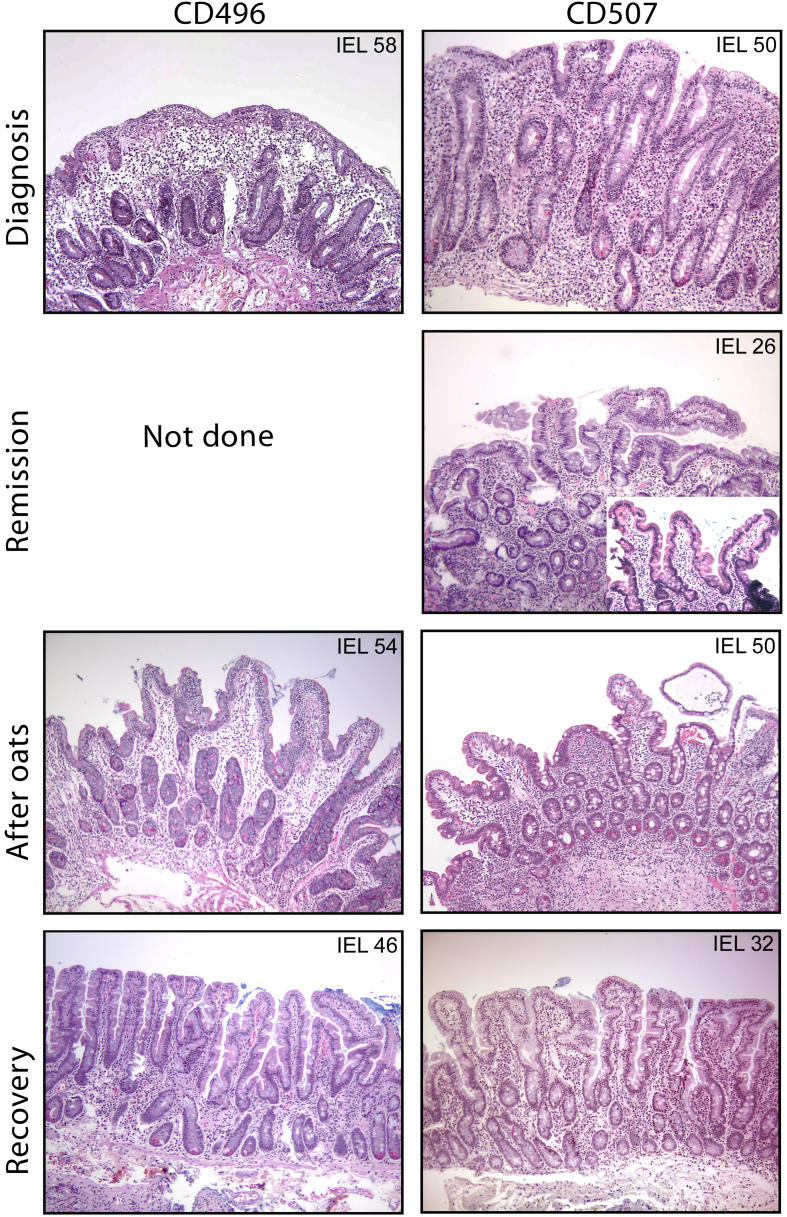
Histology of Intestinal Mucosa of Two of the Oat-Intolerant Patients Small intestinal biopsies were obtained at diagnosis, after an ordinary gluten-free diet (remission), after introduction of oats, and after withdrawal of oats (recovery). For patient CD496, a biopsy was not taken after she started with a gluten-free diet. Biopsies were scored according to the modified Marsh criteria. Hematoxilin-eosin staining was used, and IEL counts are given in the corners of the photomicrographs. The remission biopsy from patient CD507 was poorly oriented. We therefore melted and reoriented this biopsy (insert). Original magnification: 100×.

Nine adults with celiac disease who had a history of exposure to oats assessed to be free from contamination of other cereals were studied. In some cases they came for gastroduodenoscopy for clinical reasons, in other cases, they agreed to come for research reasons. The characteristics of the patients are given in Table 1. This case series is thus not a consecutive series of ordinary patients with celiac disease. Three of these patients (CD422, CD496, and CD507) were known to exhibit clinical and histopathological signs of oat intolerance. Patient CD422 developed villous atrophy and dermatitis while eating oats, and details of this patient are described elsewhere [15]. Patient CD496 was a 53-y-old woman who was evaluated for complicated celiac disease. Celiac disease was diagnosed in 1987 after 1 y with diarrhea and weight loss; a biopsy showed a Marsh 3C lesion with an intraepithelial lymphocyte (IEL) count of 58/100 enterocytes (range 53–69) (Figure 1). She responded well to a standard gluten-free diet. A control biopsy was not taken. In 2001, she started eating pure oats, but lost weight, going from 55 kg to 44 kg. While eating oats, a biopsy showed a Marsh 3A lesion with an IEL count of 54/100 enterocytes (range 43–62). The oats were discontinued, and she gradually recovered. Some months later, an intestinal biopsy demonstrated a Marsh 1 lesion with an IEL count of 46 (range 28–52). Clinically she is currently well. Patient CD507 was a 59-y-old woman who was also evaluated for complicated celiac disease. She probably had undiagnosed celiac disease since childhood and was diagnosed in 1990 after osteoporotic fractures. A biopsy showed total villous atrophy (Marsh 3C) with an IEL count of 50/100 enterocytes (range 44–54) (Figure 1). She responded well to a standard gluten-free diet. In 1999, a follow-up biopsy showed complete normalization of her mucosa (Marsh 0) with an IEL count of 26/100 enterocytes (range 24–32). In 2000, the patient started eating oats and developed bloating, abdominal pain and iron deficiency. She lost 2 kg in weight. In 2002, while still eating oats, a biopsy showed a Marsh 3A lesion with an IEL count of 50/100 enterocytes (range 38–58). She discontinued eating oats and improved clinically. A new biopsy later in 2002 showed improvement, with a Marsh 1 lesion with an IEL count 32/100 enterocytes (range 24–46). Surprisingly, in late 2003 she was diagnosed with an adenocarcinoma in the small intestine, which was removed surgically.

**Table 1:**
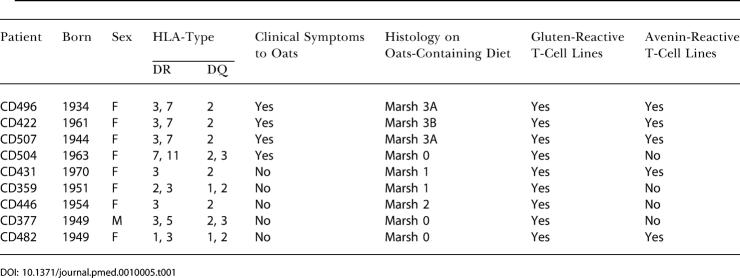
Characteristics of the Included Patients

### Avenin-Reactive T-Cell Lines Generated from Intestinal Biopsies Challenged with Avenin

Responses to TG2-treated avenin were detected in the polyclonal T-cell lines derived from the avenin-challenged biopsies from all three patients who had clinical and histopathological signs of oat intolerance (Table 1). Intestinal T-cell responses to TG2-treated avenin were also found in two of the other six patients. At least one avenin-reactive T-cell line from each patient was expanded. Inhibition experiments using anti-HLA class I and class II monoclonal antibodies demonstrated that these T-cell lines were all restricted to DQ2 (Figure 3; unpublished data), and with the exception of the T-cell line generated from patient CD482, they all gave an enhanced T-cell response to avenin treated with TG2 compared to avenin not treated with TG2 (Table 2). T-cell lines derived from the biopsies challenged ex vivo with avenin gave higher responses to the TG2-treated avenin than to TG2-treated gluten in four of five patients (CD422, CD431, CD482, and CD496, but not CD507; Table 2). Notably, intestinal T-cells specific for TG2-treated wheat gluten and gliadin were identified in control biopsies challenged with gluten in all nine celiac patients (see Table 1).

**Table 2:**
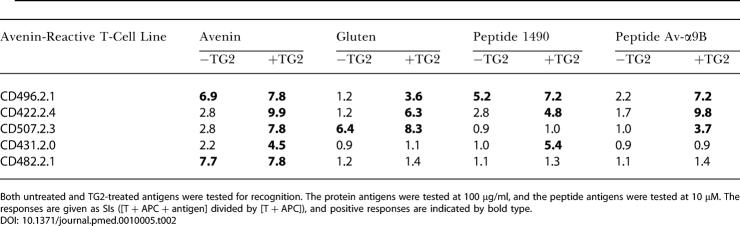
T-Cell Responses to Avenin, Gluten, and Avenin Peptides in T-Cell Lines Established from Biopsies Stimulated with Avenin Antigen

Both untreated and TG2-treated antigens were tested for recognition. The protein antigens were tested at 100 μg/ml, and the peptide antigens were tested at 10 μM. The responses are given as SIs ([T + APC + antigen] divided by [T + APC]), and positive responses are indicated by bold type

### Identification of a T-Cell Epitope in Avenin

**Figure 2:**
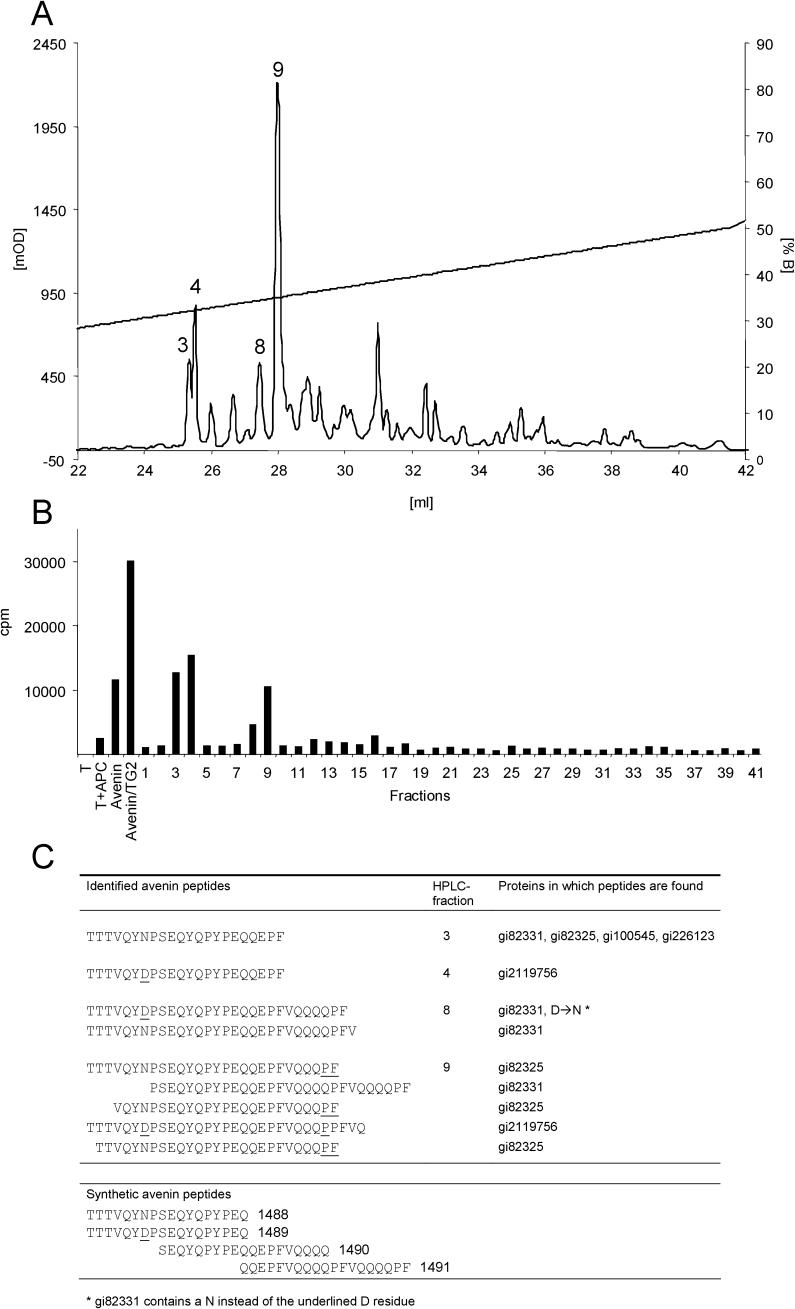
Identification of an Epitope in Avenin Recognized by Intestinal T-Cells of Celiac Disease Patients (A) Reverse-phase HPLC of a pepsin-trypsin digest of avenin. Peptides in fraction 25, obtained from gel filtration, were eluted by an acetonitrile gradient (straight line), and 41 fractions were collected. Fractions recognized by T-cell lines in subsequent experiments are indicated by the numbers above the peaks. (B) T-cell recognition of fractions obtained by reverse-phase HPLC. All 41 fractions obtained in (A) were tested for recognition by the intestinal T-cell line 422.2.4 (derived from an oat-intolerant celiac disease patient). The fractions were incubated with DR3-DQ2 homozygous antigen-presenting cells overnight before the T-cell line was added. Specific T-cell responses were measured by ^3^H-thymidine incorporation. Pepsin-trypsin-digested avenins, both TG2-treated and untreated, were used as control antigens. (C) Sequences of the peptides in the stimulatory fractions from reverse-phase HPLC identified by tandem mass spectrometry and overlapping synthetic peptides used for T-cell assays. For better comparison, the amino acid sequence of the avenin precursor protein JQ1047 (gi82331) is taken as a consensus sequence, and deviating residues are underlined.

To identify the T-cell stimulatory peptides, we initially studied an avenin-specific T-cell line (TCL CD422.2.4) isolated from the oat-intolerant patient CD422. The T-cells weakly recognized one gel filtration fraction (#25) of a pepsin-trypsin digest of avenin. This fraction was further separated by reverse-phase HPLC, and retested for T-cell recognition (Figure 2A and 2B). Stimulatory fractions were subjected to electrospray ionization tandem mass spectrometry (Figure 2C). For fractions 3 and 4, a single 22-mer peptide was identified differing only by an asparagine (fraction 3) and an aspartic acid residue (fraction 4). The two identified peptides from fraction 8 represent C-terminally elongated derivates of these 22-mers. Five peptides identified from fraction 9 correspond to N-terminally truncated and C-terminally elongated variants.

### T-Cell Recognition of Synthetic Avenin Peptides

**Figure 3:**
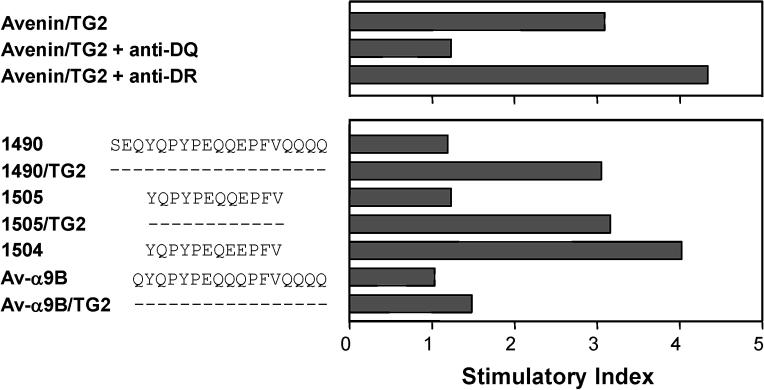
HLA Restriction and Avenin Peptide Specificity of the Intestinal T-Cell Line 431.2 from Patient CD431 The avenin antigen (at 0.25 mg/ml) and peptides (at 10 μM), treated with TG2 when indicated, were incubated overnight with DR3-DQ2 homozygous antigen-presenting cells before T-cells were added. In the HLA restriction experiments, anti-DR or anti-DQ monoclonal antibodies were added 30 min prior to the T-cells. T-cell responses were measured by ^3^H-thymidine incorporation and are represented as SIs.

**Figure 4:**
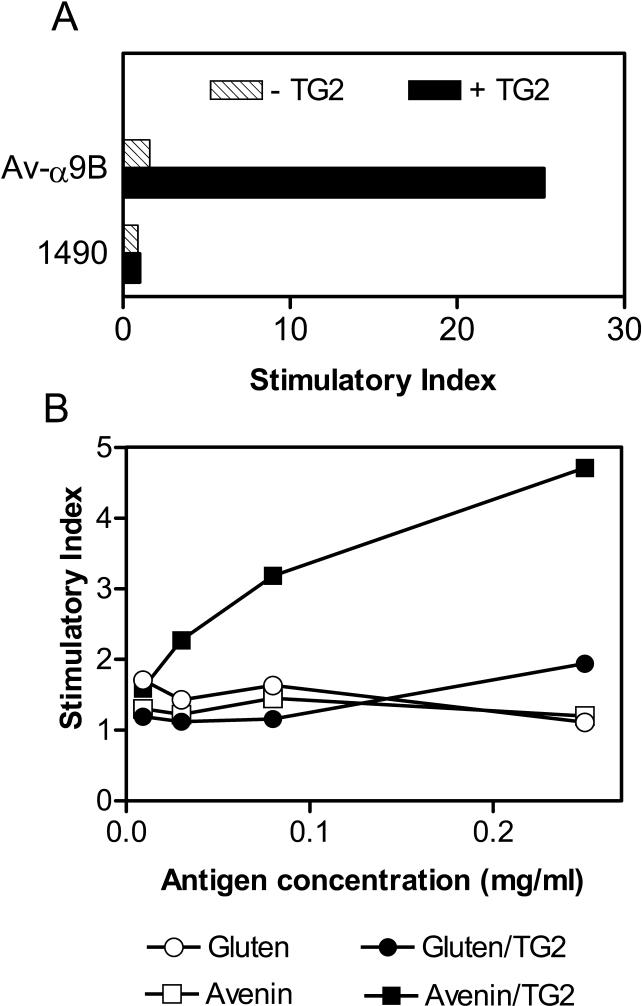
Reactivity of an HLA-DQ2-Restricted T-Cell Clone Derived from a T-Cell Line (CD496.2.3) Established by Avenin Stimulation of an Intestinal Biopsy of Patient CD496 T-cell responses were measured by ^3^H-thymidine incorporation and are represented as SIs. (A) The clone specifically recognizes the avenin peptide Av-α9B after treatment with TG2. The peptides were tested at 10 μM. (B) The clone recognizes avenin but not gluten antigen after treatment with TG2.

Four avenin peptides (1488, 1489, 1490, and 1491; Figure 2C) almost completely covering the sequences identified in the reverse-phase HPLC fractions 3, 4, 8, and 9 were synthesized and tested for T-cell recognition. Only peptide 1490 (SEQYQPYPEQQEPFVQQQQ) was recognized by the T-cell lines CD422.2.4, CD496.2.1, and CD431.2 (see Table 2; Figure 3). The recognition of this peptide by the T-cell lines CD422.2.4 and CD431.2 was dependent on TG2 treatment. T-cell line CD496.2.1 responded to the native peptide, but the response was augmented by treatment with TG2. We identified one deamidation site by tandem mass spectrometry (underlined in the above given sequence). This regioselectivity of deamidation conforms to the previously defined specificity of TG2 [11,19]. Several truncation variants of peptide 1490 were also synthesized. The shortest peptides tested were 12-mers; the T-cell line CD431.2 recognized peptide 1505 (YQPYPEQQEPFV) after TG2 treatment and the already deamidated peptide 1504 (YQPYPEQEEPFV) without TG2 treatment (Figure 3). We predict the 9-mer core region binding to DQ2 as PYPEQEEPF, placing the glutamic acid resulting from the deamidation at the P6 position. This is similar to the DQ2-α-I gliadin epitope (PFPQPELPY), which also binds to DQ2 with a glutamic acid at the P6 position [20,21]. Recently, Vader et al. studied whether T-cells generated from celiac disease biopsies stimulated with wheat gluten would cross-react with predicted epitopes of barley, rye, and oats. From these studies they found two broadly reactive polyclonal T-cell lines that responded to peptides from barley hordeins, rye secalins, and the avenin-derived peptides Av-α9A, which is identical to a length variant of 1490 (QYQPYPEQQEPFVQ), and Av-α9B (QYQPYPEQQQPFVQ) [22]. We tested peptide Av-α9B against our T-cell lines and found that it was recognized by T-cell lines from the patients CD422 (line 2.4), CD496 (lines 2.1 and 2.3), and CD507 (line 2.3) after TG2 treatment (Table 2; unpublished data). From the T-cell line CD496.2.3, we generated a T-cell clone that was specific for the peptide Av-α9B after TG2 treatment (Figure 4A). This clone responded also to TG2-treated avenin, but did not display cross-reactivity to TG2-treated gluten nor to the TG2-treated 1490 peptide (Figure 4B). The avenin-reactive T-cell line generated from the patient CD482 (CD482.2.1) did not recognize the 1490 peptide nor the Av-α9B peptide. Thus, there exist at least two distinct peptides of oats that can elicit mucosal T-cell responses in celiac disease patients with clinical intolerance to oats.

### Location of Epitopes to a Proline- and Glutamine-Rich Region of Avenin

**Figure 5:**
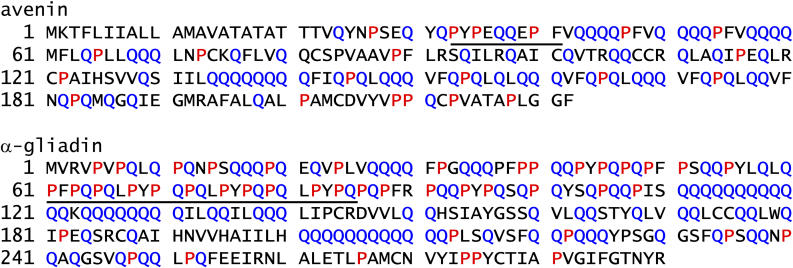
Amino Acid Sequence of an Avenin (gi 82331, JQ1047) and an α-Gliadin (α2-Gliadin, AJ133612) The proline and glutamine residues are red and blue, respectively. In the avenin, the presumed 9-mer core region of the characterized T-cell epitope is underlined. In the α-gliadin, a 33-mer natural fragment containing six copies of three partly overlapping epitopes (DQ2-α-I, PFPQPQLPY; DQ2-α-II, PQPQLPYPQ; and DQ2-α-III, PYPQPQLPY) is underlined. Note the localization of all the epitopes to regions of the proteins rich in proline and glutamine residues and the high number of proline residues within the 9-mer core regions of the epitopes.

The avenin epitopes we identified are localized to a region of avenin uniquely rich in proline and glutamine residues (Figure 5). The presumed 9-mer core region of the avenin epitopes (PYPEQQEPF and PYPEQQQPF) contains three proline residues. The high number of proline residues and the localization of the epitopes to a region rich in proline and glutamine residues bear strong resemblance to features typical of DQ2-restricted T-cell epitopes of wheat gliadin [23,24].

## Discussion

A number of previous reports concluded that all celiac disease patients tolerate oats. These reports have formed the basis for approving oats in the gluten-free diet for the treatment of celiac disease. The findings reported here demonstrate that oat intolerance exists in some celiac disease patients, and the study provides a molecular explanation for this intolerance.

Oats are less related to wheat than are barley and rye. In oats, the prolamines represent much less of the total seed proteins than in the other cereals [25]. In addition, avenins contain about half the amount of proline residues (10%) as the prolamins of wheat (gliadins and glutenins), barley (hordeins), and rye (secalines). On this basis, it is intriguing that the identified avenin epitopes are located in the regions of avenins with the highest content of proline residues, regions also rich in glutamine. This is analogous to the localization of the T-cell epitopes in α- and γ-gliadins [23]. The immunogenicity of gliadin peptides is influenced both by the glutamine residues, which become specifically deamidated by TG2, and by the proline residues, which protect the peptides from proteolysis in the gastrointestinal tract, determine the specificity of TG2, and are crucial for the selective binding to HLA-DQ2 [1]. This study shows that the same features apply to T-cell epitopes of avenin.

In humans it is impossible to directly demonstrate that T-cells induce disease. In celiac disease this relates equally to T-cells reactive to gluten and to T-cells reactive to avenin. The fact that avenin-reactive intestinal T-cells, like gluten-reactive T-cells from celiac disease patients, are uniquely restricted by HLA-DQ2 and are activated by TG2-treated peptides speaks strongly in favor of their involvement in the disease pathogenesis. The finding of avenin-specific intestinal T-cells also in individuals with celiac disease that are clinically tolerant to oats does not, as we see it, contradict this assumption. Some patients with celiac disease stay in remission for extended time periods during gluten challenge even if it is likely that they have gluten-reactive T-cells in their intestinal mucosa. Since avenin is less immunogenic than wheat gluten, one would expect an extended time for relapse to be at least as common during oats consumption.

It is highly unlikely that the intolerance and the mucosal inflammation observed in our patients could be explained by contamination of the oat flour by wheat, barley, or rye proteins. All the oats consumed were produced in a quality-assessed production line. Our data indicate that avenin can drive mucosal inflammation in that the incubation of the intestinal biopsies with avenin enriches for activated, avenin-reactive T-cells. A substantial proportion of the avenin-reactive T-cells appear to be specific to avenin. The T-cell clone we established from an avenin-challenged biopsy was reactive to avenin but did not cross-react to wheat gluten, and the T-cell lines from biopsies challenged with avenin responded more strongly to avenin than to gluten in four of five participants. Cross-reactivity at the T-cell clonal level has been demonstrated between wheat gluten, hordein, and secalin antigens [22,26] and likely also exists between gluten and avenin [22]. Even if some of the avenin-reactive T-cells were originally primed to gluten and responded to avenin because of cross-reactivity, they would still participate in an avenin-driven immune response.

T-cell reponses to the avenin epitopes described in this paper have been found in T-cell lines derived from intestinal biopsies of patients with celiac disease that were stimulated with gliadin [22]. It is unknown whether any of the patients from whom these T-cells were isolated had clinical symptoms or mucosal inflammation related to oats ingestion. Thus, to our knowledge, the current study is the first to demonstrate a mechanistic link between clinical symptoms of oat intolerance, mucosal inflammation, and avenin-reactive T-cells.

Oat intolerance can cause complications in the large group of celiac disease patients who are now regularly consuming oats. At this stage we do not know how frequently such complications may occur. Presumably such complications will not be very common, but only extended clinical follow-up of oats-consuming celiac disease patients will establish the frequency. Monitoring of T-cell responses to avenin epitopes may potentially identify individuals who are at risk of developing oat intolerance. Based on our data, such monitoring will also identify some individuals who are clinically tolerant to oats and who have minimal or no mucosal pathology after a limited oats challenge. Possibly some of these patients may have latent oat intolerance that will develop into overt disease after prolonged exposure, but this remains speculative. Our observations demonstrate that even if oats seem to be well tolerated by many celiac disease patients, there are patients who have an intestinal T-cell response to oats. Until the prevalence of oat intolerance in celiac disease patients is established, clinical follow-up of celiac disease patients eating oats is advisable. Clinicians should be aware that oat intolerance may be a reason for villous atrophy and inflammation in patients with celiac disease who are eating oats but otherwise are adhering to a strict gluten-free diet.

**Patient Summary**

**Background**

Celiac disease is a digestive disease that damages part of the gut (the small intestine) and interferes with absorption of nutrients from food. Patients with celiac disease do not tolerate a protein called gluten, which is found in wheat, rye, and barley. When people with celiac disease eat foods containing gluten, their immune system responds by damaging the small intestine. The disease is quite serious in some patients, but eating a strictly gluten-free diet can eliminate all of the symptoms. Unfortunately, wheat, barley, and rye products like flour are found in many common foods, and patients have to avoid them for the rest of their lives. Previous studies suggested that oats were safe for patients with celiac disease, and as a result, they often form part of a gluten-free diet.

**What Did the Researchers Find?**

Contrary to other studies, this one demonstrates that oats intolerance does exist in some patients with celiac disease. These patients have an immune reaction to oats that is similar to the reaction most celiac disease patients have to wheat, barley, and rye.

**What Does This Mean for Patients?**

It appears that oats are not safe for all patients with celiac disease. Patients who eat oats as part of a gluten-free diet should discuss their diet and any symptoms with their doctors; doctors should keep in mind that patients might develop symptoms when they eat oats.

**What Are the Problems with the Study?**

The researchers studied only a small number of patients, and this study cannot tell us how common oats intolerance is among celiac disease patients.

**Where Can I Find More Information?**

US National Institutes of Diabetes, Digestive, and Kidney Disorders: http://digestive.niddk.nih.gov/ddiseases/pubs/celiac/

Celiac Disease Foundation: http://www.celiac.org/

The Gluten Intolerance Group: http://www.gluten.net/

The Celiac Disease Foundation: http://www.celiac.com/

## Abbreviations

IEL: intraepithelial lymphocyte
SI: stimulatory index
TG2: tissue transglutaminase
